# Supplementary material for: Sociodemographic predictors of the association between self-reported sleep duration and depression
Source: PLOS Glob Public Health. 2024 Jun 12;4(6):e0003255. doi: 10.1371/journal.pgph.0003255 (PMC11168698; doi:10.1371/journal.pgph.0003255)
Supplement: S1 Table — MS: Marital Status, ES: Employment Status. (DOCX) [file pgph.0003255.s001.docx]

| S1 Table. Estimated odds ratios (95% CIs), z-value, p-value from 100 multiple imputations using Rubin’s rules. MS: Marital Status, ES: Employment Status. | | | |
| --- | --- | --- | --- |
| Variables | OR (95% CI) | z-value | p-value |
| BMI | 0.989 (0.981, 0.996) | -2.856 | 0.004 |
| age | 0.981 (0.974, 0.989) | -4.683 | <0.0001 |
| Gender | 1.033 (0.928, 1.149) | 0.585 | 0.558 |
| Total PHQ-8 | 0.966 (0.957, 0.976) | -6.687 | <0.0001 |
| ES - Employed | Reference group | | |
| ES - Unemployed | 1.334 (1.161, 1.533) | 4.075 | <0.0001 |
| ES - Students | 1.262 (1.083, 1.47) | 2.979 | 0.003 |
| MS - Married | Reference group | | |
| MS - Others | 1.306 (0.977, 1.745) | 1.804 | 0.071 |
| MS - Single | 1.033 (0.912, 1.171) | 0.516 | 0.606 |
