# Supplementary material for: Sociodemographic predictors of the association between self-reported sleep duration and depression
Source: PLOS Glob Public Health. 2024 Jun 12;4(6):e0003255. doi: 10.1371/journal.pgph.0003255 (PMC11168698; doi:10.1371/journal.pgph.0003255)
Supplement: S1 Text — (DOCX) [file pgph.0003255.s002.docx]

**Binarization of the PHQ-8 Score: Enhancing Depression Screening and Assessment**

The Patient Health Questionnaire-8 (PHQ-8) is a widely used self-report tool designed to screen and assess the severity of depression. It consists of eight questions that inquire about various depressive symptoms experienced over the past two weeks. Traditionally, the PHQ-8 score has been used as a continuous measure to gauge the severity of depression. Yet, there has been growing interest in binarizing the PHQ-8 score to simplify its interpretation and enhance its clinical utility.

However, in some clinical and research contexts, a simpler binary classification of depression presence or absence can be more practical. This process is referred to as the "binarization" of the PHQ-8 score. Binarization involves setting a threshold score, above which an individual is classified as experiencing depression, and below which they are not. The binarized PHQ-8 score thus converts a continuous measure of depression severity into a categorical outcome, which can facilitate quick screening and decision-making.

Threshold of ten is often used as it balances sensitivity and specificity reasonably well. Individuals with PHQ-8 scores of 10 or higher are classified as potentially having depression. This threshold is based on clinical research and has been widely implemented in primary care settings.

Binarization of the PHQ-8 score has its benefits as binarization simplifies the interpretation of the PHQ-8 score, making it more accessible to healthcare professionals and individuals undergoing screening. Furthermore, binarization enables quicker assessment of depression presence, which can be valuable in time-sensitive clinical settings. Moreover, binarized scores can aid in directing resources and interventions to those most likely to benefit.

However, binarization of the PHQ-8 score has also its limitations as converting a continuous score to a binary outcome sacrifices the ability to differentiate between varying degrees of depression severity. And binarization can, in many cases, result in false positives or negatives, depending on the chosen threshold, impacting the accuracy of screening. Furthermore, depression is a complex condition with multiple symptom presentations; binarization oversimplifies this complexity.

In conclusion, the binarization of the PHQ-8 score can be a valuable tool in clinical practice, offering a simplified and actionable interpretation of depression severity. However, it's important to recognize that the binary classification does not capture the full spectrum of depression experiences, and it should be used in conjunction with clinical judgment and comprehensive assessment. Clinicians should also remain open to the possibility of adjusting the threshold based on ongoing research and the evolving understanding of depression diagnosis and treatment.

References:

- Razykov I, Ziegelstein RC, Whooley MA, Thombs BD. The PHQ-9 versus the PHQ-8—is item 9 useful for assessing suicide risk in coronary artery disease patients? Data from the Heart and Soul Study. Journal of psychosomatic research. 2012 Sep 1;73(3):163-8.
- Kroenke K, Strine TW, Spitzer RL, Williams JB, Berry JT, Mokdad AH. The PHQ-8 as a measure of current depression in the general population. Journal of affective disorders. 2009 Apr 1;114(1-3):163-73.
