# Supplementary material for: Sociodemographic predictors of the association between self-reported sleep duration and depression
Source: PLOS Glob Public Health. 2024 Jun 12;4(6):e0003255. doi: 10.1371/journal.pgph.0003255 (PMC11168698; doi:10.1371/journal.pgph.0003255)
Supplement: Attachment — Submitted filename: Response to Reviewers.docx [file pgph.0003255.s003.docx]

**Response to Reviewers**

**Point by point authors’ response to editor’s review:**

We thank the editor for the review and this assessment of the submitted manuscript. We accept all the suggestions made by the editor’s revision which actually has improved the over all content and structure of manuscript. This letter summarizes the authors’ response to the constructive comments and suggestions made by the editor.

Kindly note that we highlighted the edits in the revised manuscript which is labeled “Revised Manuscript with Track Changes”. The changes on previous submitted manuscript are “tracked” for your review. Also, the texts, tables, figures with red color font are newly added since results, discussion and conclusion parts needed to be fully edited based on the statistical analysis and sensentivity analysis which was done after editor review. Based on that references were updated. Whilst the black color font text had no changes done on it, so kept black.

The reviewer commented the following:

1.      **"** **Structure of manuscript:
Consider to add a conclusion section of the full-text manuscript in consistency with the abstract at the beginning was written.**

***Authors response,***

We thank the reviewer for the feedback and we agree with the comment. Therefore, a conclusion section of the full-text manuscript is added based on the statistical analysis and sensentivity analysis of revised manuscript.

Kindly refer to line **300**.

**2. Title of manuscript
Look at the aim/Objective of the abstract and full-text seems quite different and not consistent. For example, in the abstract was "to investigate the relationship between self-reported sleep hours and the eight items 22 patient health questionnaire (PHQ-8) as a screening measure for depression among Emirati adults" but in the Introduction section was "to investigate the association between self-perceived sleep duration and depression considering larger sample size and further factors such as sociodemographic factors such as marital status, employment status after adjusting for age and gender". It means authors need to revise the title how to fit what this cross sectional study expect to achieve, not only to investigate the association between sleep duration and depression.**

***Authors response,***

We thank the reviewer for the comment. Kindly note the change has been done and the title of the manuscript was rephrased. Also similar sentence was mentioned at the end of the introduction section. Therefore, the title and the above sentense were rephrased.

Kindly refer to line **1** in title, line **23** in the abstract and line **137** in the introduction section.

3**. "Study design
Authors have to make sure this study is mainly cross sectional study design and the data set used was a Baseline data from 11,455 UAE cohort Study. Therefore, authors need to see the overall manuscript to make it clearer in writing.**

***Authors response,***

We thank the reviewer and we agree with the comment. For that kindly see the little changes done to reflect this study is mainly cross sectional and the data set used was a baseline data from 11,455 UAE cohort Study. Also, this was written clearer in other sections.

Kindly refer to line **26** in abstract, line **139** in introduction section and line **148** materials and methods section.

**4. " Introduction section
It's very unclear that which variable is cause and which variable is effect in their relationship analysis. As seen in the findings, sleep duration is dependent variable and depression plus other predictors (sociodemographic factors and BMI) are independent variable. Therefore, it's very necessary to provide more stronger evidences from literature review to confirm sleep disorder is effect (outcome variable) in this introduction section. Some of studies also mentioned that sleep disorder can be a risk factors of depression.**

***Authors response,***

We thank the reviewer for the feedback. We agree and accept all these comments. Kindly note this section was meant to be edited prior the initial submission. Therefore, we apologize for the confusion caused. The edits were done and it is made clear that, the self-perceived sleep duration is the outcome and other variables are predictors (total PHQ-8, adjusted for age, gender, BMI, marital status, and employment status). Moreover, based on that, the necessary statistical analysis, results, tables, figures, discussion and conclusion section were all updated.

Kindly refer to the lines from **47** to **139** in introduction section.

**5. " Measures section
Sleep duration, it is better to apply it as continuous variable and it's actually more valuable than sleep duration is classified as categories (qualitative variable), in multivariate regression analysis (OR can  not be applicable in this manuscript). Otherwise, authors can apply OR analysis but sleep duration is classified as Binary variable (Normal/sleep disorder) is the best. This comment is quite open for authors to consider for revision.**

***Authors response,***

Thank you for the comment. We totally agree that a multivrariate regression analysis should be used. Therefore, to examine the factors associated with self-reported sleep, univariate and multivariate logistic regression models were performed with the dichotomized self-reported sleep as the outcome. The predictors were Total PHQ-8 (linear), Age (linear), Gender (Females vs. Males), BMI (linear), and Marital status (categorical), employment status (categorical). Moreover, a multivariate imputation by chained equations (MICE) procedure was applied with classification and Regression Trees (CART) to impute missing values.

The changes has been done accordignally and the sentences were rephrased.

Kindly refer to line **164** in statistical analysis section, sensetitivity analysis in line **175** and (**Table 1 in line 199 & Table 2 in line 215, Figure 1 in line 222 and Figure 2 in 251**).

**In generl, we thank the editor for the over all comments as we feel these amendments have majorly improved the academic value and content of our work.**

**Thank you**

**Corresponding author**
