# Supplementary material for: Sociodemographic predictors of the association between self-reported sleep duration and depression
Source: PLOS Glob Public Health. 2024 Jun 12;4(6):e0003255. doi: 10.1371/journal.pgph.0003255 (PMC11168698; doi:10.1371/journal.pgph.0003255)
Supplement: Attachment — Submitted filename: Rebuttal letter.docx [file pgph.0003255.s005.docx]

**Rebuttal letter**

**This rebuttal letter includes point by point authors’ response to reviewers’ comments and academic editors since the initial submission up to recent letter of 18^th^ April:**

First of all, authors would like to thank the reviewers and the academic editors for their valuable reviews and comments which helped to indicate the areas of the manuscript that needed to be improved and dynamically upgraded the manuscript structure, content and overall representativeness.

**Point by point response to Academic Editor and Reviewers for the recent letter dated 18^th^ April 2024**

**1. Academic Editor comments:** Journal Requirements:

Please review your reference list to ensure that it is complete and correct. If you have cited papers that have been retracted, please include the rationale for doing so in the manuscript text or remove these references and replace them with relevant current references. Any changes to the reference list should be mentioned in the rebuttal letter that accompanies your revised manuscript. If you need to cite a retracted article, indicate the article’s retracted status in the References list and also include a citation and full reference for the retraction notice.

***Authors response,***

We thank the editor for their review and comments. We accept their comments and value its importance for reflecting correct and complete reference list in relation to the content of the paper. We as well revised the journal referencing style and criteria. We reviewed every single reference and its related citations within the text of the manuscript.

Therefore, the following amendments have been made, kindly refer to references in the **revised article with changes highlighted** in line starting 598: ***The references*** which are highlighted with green, were amended to the Vancouver style to ensure following the PLOS Global Public Health Journal Guidelines. While the references highlighted with Blue took place over retracted ones. Also, references that are highlighted with yellow are added to the references list in order to support with strong evidenced based literature and practices compared to removed ones. Also, references highlighted in gray, were deleted from the paper. However, the references with no highlight (with black text) and those with the previously tracked changes, are those which weren’t amended.

Moreover, relative changes for references numbers within manuscript text were amended accordingly.

**Figures:**

Finally, Kindly note that figures were uploaded to the PLOS Preflight Analysis and Conversion Engine (**PACE**) digital diagnostic tool, ***successfully*.**

**2. Reviewer’s comments**

Thanks for your patient to take a lot of time for very serious revision of manuscript. We re-consider your latest manuscript was revised carefully upon reviewers' comments on research methodology (clearer variable clarification and statistical data analysis...). It's very much appreciated with stronger evidence of the findings of study on "Sociodemographic predictors of the association between self-reported sleep duration and depression". Before we will make a final decision for possible publication, the author should add a conceptual framework of your study, in your Measures section of the full text, to clarify better the cause-effect relationship between study variables (The predictors of "self-reported sleep hours" variable are the self-reported depression and other factors as described).

***Authors response,***

We thank the reviewer for the valuable feedback, and we agree with the comment that forming a conceptual framework for the outcome and predictors will clarify better the cause-effect relationship between study variables. Therefore, Figure 1 is added to the measures section in the **revised article with changes highlighted**, line 203. Also, a legend for figure 1 is added to the supporting information in line 873. Moreover, other figures (2&3) order have been amended within the text of manuscript and in the legend of the supporting information of the in the **revised article with changes highlighted**.

***We thank the Academic Editor and reviewer for their valuable feedback. We believe the amendments made based on their comments were crucial for improving the academic value and content of our work.***

***Corresponding author***

***18th April 2024***

**Rebuttal letter for previous revisions since original submission from 3^rd^ August 2023**

**to 5^th^ April 2024.**

The reason I am requesting this appeal is due to the recent comments from reviewers which were on 31st January 2024. I am in full respect and agree with all their comments which already has been considered previously and some recent ones could be modified to meet their comments. I believe some reviewers’ comments are on the initial submission version and are not on the revised manuscript which was submitted on 12th October 2023, and they looked at the initial one which was on 3rd August 2023. Also, based on their recent comments, a round of revision could be achieved instead of complete rejection since few modifications could meet their valuable comments.

**History of this manuscript submission**

Kindly note the initial submission was submitted on 3^rd^ August 2023. Then Editor sent to author the need for technical check. As a response to that, the corresponding author has attached the required documents, Human subject research checklist and the ethical approval of the study. Then, on September 10^th^, the editor sent a decision for a minor revision based on reviewers’ comments for the initial submission. You can find below the authors’ point by point response to reviewers’ comments as was submitted on 12^th^ October 2023. A week later, other comments were sent by Editors. As a response to that, Author submitted a revised version on 1^st^ November 2023. Two months later, the author received a rejection decision although reviewers and academic editors’ comments were all addressed by authors.

**Kindly find below the point-to-point responses starting from the initial submission on 3^rd^ August until latest reviewers’ comments on January 2024. All over this letter which contains all three rounds of point-by-point response to reviewers' comments. Kindly note all responses are connected to the *(revised article with changes highlighted).* Moreover, in order to make it more convenient to the reviewers/editors, all the parts of the paper which appear in “Grey highlight”, these indicates removed or replaced sections as a response to reviewers’ comments. Finally, the text in blue indicates the author’s response to reviewers’ comments.**

1. **Point by point author’s response to reviewer comments.**

**A revised manuscript with tracked changes was submitted on 12^th^ October 2023**

Kindly note responses here are based on the edits which are revised-highlighted version with tracked changes. Whilst the black color font text had no changes done on it, so kept black.

Reviewer commented the following:

**1.a Reviewer/editor comment****:**

***Structure of manuscript: Consider adding a conclusion section of the full-text manuscript in consistency with the abstract at the beginning was written.***

***Authors response,***

We thank the reviewer for the feedback, and we agree with the comment. Therefore, a conclusion section of the full-text manuscript is added based on the statistical analysis and sensitivity analysis of revised manuscript.

Kindly refer to line 506 **in the Revised Article with Changes Highlighted.**

**1.b Reviewer/editor comment:**

***Title of manuscript: Look at the aim/Objective of the abstract and full text seems quite different and not consistent. For example, in the abstract was "to investigate the relationship between self-reported sleep hours and the eight items 22 patient health questionnaire (PHQ-8) as a screening measure for depression among Emirati adults" but in the Introduction section was "to investigate the association between self-perceived sleep duration and depression considering larger sample size and further factors such as sociodemographic factors such as marital status, employment status after adjusting for age and gender". It means authors need to revise the title how to fit what this cross-sectional study expects to achieve, not only to investigate the association between sleep duration and depression.***

***Authors response,***

We thank the reviewer for the comment. Kindly note the change has been made and the title of the manuscript was rephrased. Also, related edits done on the sentence at the end of the introduction section (line 155) **in the Revised Article with Changes Highlighted**. Also, related necessary amendments were applied throughout the measures, discussion, and conclusion sections.

Kindly refer to the edited abstract, measures in line 172, in line 419 discussion, and line 506 in the conclusion sections of **the Revised Article with Changes Highlighted,** for the changes done in which it reflects the aim and objectives of the cross-sectional study.

**1.c Reviewer/editor comment:**

***Study design: Authors have to make sure this study is mainly a cross-sectional study design and the data set used was a Baseline data from 11,455 UAE cohort Study. Therefore, authors need to see the overall manuscript to make it clearer in writing.***

***Authors response,***

We thank the reviewer, and we agree with the comment. For that kindly see the little changes done to reflect this study is mainly cross sectional and the data set used was a baseline data from 11,455 UAE cohort Study.

Kindly refer to line 39 in abstract, line 169 in the methods, line 255 in the results section and all over the tables in the statistical analysis section **in the Revised Article with Changes Highlighted**.

**1.d Reviewer/editor comment:**

***Introduction section: Its very unclear which variable is cause and which variable is effect in their relationship analysis. As seen in the findings, sleep duration is dependent variable and depression plus other predictors (sociodemographic factors and BMI) are independent variable. Therefore, it's very necessary to provide stronger evidence from literature review to confirm sleep disorder is effect (outcome variable) in this introduction section. Some studies also mentioned that sleep disorder can be a risk factor of depression.***

***Authors response,***

We thank the reviewer for the feedback. We agree and accept all these comments. Kindly note this section was meant to be edited prior to the initial submission. Therefore, we apologize for the confusion caused. The edits were done, and it is made clear that the self-perceived sleep duration is the outcome and other variables are predictors (total PHQ-8, adjusted for age, gender, marital status, and employment status).

Kindly refer to the lines from **67 to 105** in the introduction section **in the Revised Article with Changes Highlighted**. Moreover, based on that, the necessary statistical analysis, results, tables, figures, discussion, and conclusion section were all updated.

**1.e Reviewer/editor comment:**

***Measures section: Sleep duration, it is better to apply it as continuous variable and it's actually more valuable than sleep duration is classified as categories (qualitative variable), in multivariate regression analysis (OR cannot be applicable in this manuscript). Otherwise, authors can apply OR analysis, but sleep duration is classified as Binary variable (Normal/sleep disorder) is the best. This comment is quite open for authors to consider for revision.***

***Authors response,***

Thank you for the comment. We totally agree that a multivariate regression analysis should be used. Therefore, to examine the factors associated with self-reported sleep, univariate and multivariate logistic regression models were performed with the dichotomized self-reported sleep as the outcome. The predictors were Total PHQ-8 (continues), Age (continues), Gender (Females vs. Males), BMI (continues), and Marital status (categorical), employment status (categorical). Moreover, a multivariate imputation by chained equations (MICE) procedure was applied with classification and Regression Trees (CART) to impute missing values.

The changes have been made accordingly and the sentences were rephrased These are newly added since results, discussion and conclusion parts needed to be edited based on the statistical analysis and sensitivity analysis which was done after editor review. Based on that references were updated. Kindly refer to statistical analysis section line 217 and sensitivity analysis line 241 **in the Revised Article with Changes Highlighted**. Also, the texts, tables, figures highlighted with this color shade.

In regard to treating sleep duration as a continues variable, please refer to manuscript line 181, and Figure 2 in line 323 where sleep duration was also measured as a continues variable. Line 181: “Sleep duration was dichotomized into two sleep groups (sleep >7 and sleep <6), this is due to our expectation to have wide variation of sleep duration which starts from 3 hours per pay up to 21 hours per day since having variation in the individuals’ sociodemographic factors [48,49]. Similar research used it as two groups [69,70]. However, self-reported sleep duration was also analyzed as a continuous variable.” Please see discussion part in which this point was highlighted in line **459 in the Revised Article with Changes Highlighted**. Kindly note the edits done in table 1 (line 265) where it is replaced with “Extreme” phrase. Also, see references as below:

**References:**

- Sheehan CM, Frochen SE, Walsemann KM, Ailshire JA. Are US adults reporting less sleep? Findings from sleep duration trends in the National Health Interview Survey, 2004–2017. Sleep. 2019 Feb;42(2): zsy221.

- Abe T, Aoki T, Yata S, Okada M. Sleep duration is significantly associated with carotid artery atherosclerosis incidence in a Japanese population. Atherosclerosis. 2011 Aug 1;217(2):509-13.

- Hirshkowitz, M., Whiton, K., Albert, S.M., Alessi, C., Bruni, O., DonCarlos, L., Hazen, N., Herman, J., Katz, E.S., Kheirandish-Gozal, L. and Neubauer, D.N., 2015. National Sleep Foundation’s sleep time duration recommendations: methodology and results summary. Sleep Health, 1(1), pp.40-43.

- Chambon, S., Galtier, M. N., Arnal, P. J., Wainrib, G., & Gramfort, A. (2018). A deep learning architecture for temporal sleep stage classification using multivariate and multimodal time series. IEEE Transactions on Neural Systems and Rehabilitation Engineering, 26(4), 758-769.‏

Thank you.

Corresponding author

2. **Point by point author’s response to Academic Editor**

**& revised manuscript re-submitted with tracked changes on 1^st^ November 2023**

**Academic Editor comment:**

***Thank you for submitting the above manuscript to PLOS ONE. During our internal evaluation of the manuscript, we found a significant text overlap between your submission and the following previously published works, some of which you are an author.*** [*https://bmcpsychology.biomedcentral.com/articles/10.1186/s40359-022-00940-3*](https://bmcpsychology.biomedcentral.com/articles/10.1186/s40359-022-00940-3)***. We would like to make you aware that copying extracts from previous publications, especially outside the methods section, word-for-word is unacceptable. In addition, the reproduction of text from published reports has implications for copyright that may apply to the publications. Please revise the manuscript to rephrase the duplicated text, cite your sources, and provide details as to how the current manuscript advances on previous work. Please note that further consideration is dependent on the submission of a manuscript that addresses these concerns about the overlap in text with published work.***

**Author’s response:**

We thank the editors for their valued comments on the revised manuscript. The editor raised a crucial issue to the submitted manuscript. Therefore, the authors have rewritten the background in the abstract section. Please refer to background section in line 24. Moreover, the authors have rewritten some parts of the introduction section in order to avoid reproduction of text previous author’s published reports. Please refer to lines 67 until 105 and 155 **in the Revised Article with Changes Highlighted**.

Thank you.

Corresponding author

3. **Point by point author’s response to reviewers based on the decision made on 31^st^ January 2024.**

We thank the reviewer for this comprehensive assessment of the submitted manuscript. We have looked carefully into the comments and this letter summarizes the authors’ response to the constructive comments and suggestions for revising the manuscript.

Reviewer#1 raised the following four crucial issues with the original manuscript. The authors have tried to answer the reviewer’s comments, point by point below.

**3.a. Reviewer #1 comment:**

***The aim of the study is to “investigate the relationship between self-reported sleep hours and the eight items patient health questionnaire (PHQ-8) as a screening measure for depression among Emirati adults”. However, I am not sure the authors achieved this aim.***

**Authors response:**

We thank the reviewer for the careful review; we agree with the comments, however we have received a similar comment from a previous reviewer comment during the initial revision cycle and a point-to-point response was submitted on 12th October 2023.

Kindly note the change has been made in the different sections of the manuscript in order to bring dynamic association between aim and the paper. Please refer to section 1.b, 1.c and 1.D within this letter. Moreover, please refer to abstract conclusion section in line 47 and paper discussion in line 402 and conclusion section in line 487 **in the Revised Article with Changes Highlighted**. Also, related required edits were applied in different Please see the aim and the conclusion as below.

The aim of the study as following: “In this cross-sectional study, we investigate the association between self-perceived sleep duration as an outcome and self-perceived depression as a predictor, after adjustment for sociodemographic factors as age, gender, marital status, employment status, in a large UAEHFS cohort.”

The conclusion of the findings as following: “The study findings indicate a correlation between self-reported depression and an increased probability of individuals reporting shorter self-perceived sleep durations especially when considering the sociodemographic factors as predictors. There was a variation in the effect of depression on sleep duration among different study groups. In association between reported sleep duration and reported depression, students and unemployed individuals have reported longer sleep hours as compared to employed participants. Also, married people reported a higher percentage of longer sleep duration as compared to single and unmarried ones when examined reported depression as a predictor to sleep duration. However, there was no difference in gender self-perceived sleep duration when associated with reported depression.”

**3.b. Reviewer #1 comment:**

***- I don’t understand why the authors did not choose to treat sleep duration as a continuous measure.***

***--In particular, I feel like the statistical methods were overly complicated and poorly justified. Given that previous research has found correlations between depression and both too little and too much sleep, I would have like to have seen a regression or at least a scatter plot of PHQ-8 score and sleep hours, as I imagine the relationship may have been u shaped (this is indicated in the box blots the authors included).***

***-If there was a good reason for grouping the sleep categories in the way they did, I would have needed more justification for this to make sense.***

***-In any case, I find multinomial regression extremely complicated to explain and I generally try to avoid it. Given this data and the authors’ aims, I would have thought some sort of linear or non-parametric regression would be easier to explain. If the hypothesis is that the relationship between depression and sleep is moderated by age/gender/BMI, stratified analysis or the inclusion of an interaction term would have made more sense.***

**Authors response:**

- **As reviewer’s commented that *“I don’t understand why the authors did not choose to treat sleep duration as a continuous measure “.***

We thank the reviewer for the comment. However, kindly note, this comment was met previously in the initial revision cycle and a response to reviewers’ letter was submitted with the edits done. Please see section 1.e. You can also refer to manuscript line 181, and Figure 2 in line 323 where sleep duration was also measured as a continues variable **in the Revised Article with Changes Highlighted**.

**References:**

- Sheehan CM, Frochen SE, Walsemann KM, Ailshire JA. Are US adults reporting less sleep? Findings from sleep duration trends in the National Health Interview Survey, 2004–2017. Sleep. 2019 Feb;42(2): zsy221.

- Hirshkowitz, M., Whiton, K., Albert, S.M., Alessi, C., Bruni, O., DonCarlos, L., Hazen, N., Herman, J., Katz, E.S., Kheirandish-Gozal, L. and Neubauer, D.N., 2015. National Sleep Foundation’s sleep time duration recommendations: methodology and results summary. Sleep Health, 1(1), pp.40-43.

- ***Also, as reviewer commented that “If there was a good reason for grouping the sleep categories in the way they did, I would have needed more justification for this to make sense.”***

We thank the reviewer for the comment. However, kindly note, this comment was met previously in the initial revision cycle and a response to reviewers’ letter was submitted with the edits done. Please see section 1.e.

Withing the manuscript, please see line 181 **in the Revised Article with Changes Highlighted**: “Sleep duration was dichotomized into two sleep groups (sleep >7 and sleep <6), this is due to our expectation to have wide variation of sleep duration which starts from 3 hours per pay up to 21 hours per day since having variation in the individuals’ sociodemographic factors [48,49]. Similar research used it as two groups [69,70]. However, self-reported sleep duration was also analyzed as a continuous variable.”

The method of dividing sleep duration into binary groups – those who report less than 7 hours and those who report 7 hours or more – is a widely accepted approach in sleep research. Recommendations from numerous health organizations are incorporated into this binary classification. For example, the National Sleep Foundation advocates 7 to 9 hours of sleep for adults to maintain optimal health. This suggests that at least 7 hours is generally considered a crucial threshold for adequate sleep, supporting its use as a cutoff point in sleep studies [71,72]. However, a multivariate linear regression model was also used to analyze sleep duration as continuous variable (Figure 1) **in the Revised Article with Changes Highlighted**.

**References:**

- Chambon, S., Galtier, M. N., Arnal, P. J., Wainrib, G., & Gramfort, A. (2018). A deep learning architecture for temporal sleep stage classification using multivariate and multimodal time series. *IEEE Transactions on Neural Systems and Rehabilitation Engineering,* 26(4), 758-769.‏

- Hirshkowitz, M., Whiton, K., Albert, S.M., Alessi, C., Bruni, O., DonCarlos, L., Hazen, N., Herman, J., Katz, E.S., Kheirandish-Gozal, L. and Neubauer, D.N., 2015. National Sleep Foundation’s sleep time duration recommendations: *methodology and results summary. Sleep Health*, 1(1), pp.40-43.

- Watson, N.F., Badr, M.S., Belenky, G., Bliwise, D.L., Buxton, O.M., Buysse, D., Dinges, D.F., Gangwisch, J., Grandner, M.A., Kushida, C. and Malhotra, R.K., 2015. Recommended Amount of Sleep for a Healthy Adult: A Joint Consensus Statement of *the American Academy of Sleep Medicine and Sleep Research Society*. Sleep, 38(6), pp.843-844.

***-As the reviewer also commented that “In any case, I find multinomial regression extremely complicated to explain and I generally try to avoid it. Given this data and the authors’ aims, I would have thought some sort of linear or non-parametric regression would be easier to explain. If the hypothesis is that the relationship between depression and sleep is moderated by age/gender/BMI, stratified analysis or the inclusion of an interaction term would have made more sense.”***

We thank the reviewer for the comment, however, also please note that this comment was also addressed by a previous reviewer comment. Please see section 1.e in this letter.

Additionally, please note that the revised manuscript which included three tables and two figures which represents the aim of the paper and were all mentioned in the results section and illustrated in the discussion and conclusion sections to meet the aim of the paper which is investigating the relationship between sleep duration and depression adjusting for sociodemographic factors, please see as follow:

- For the reviewer’s convenience, we listed them as following **in the Revised Article with Changes Highlighted**:

line 265 in **Table 1.** Number and percentages of the categorical variables by sleep categories. Chi square p-values were computed for categorical variables and Wilcoxon rank sum test for continues variables.

Line 282 in **Table 2**. Estimated odds ratios (95% Cis), z-value, p-value from fitted univariate and multivariate logistic regression model associated with self-reported Sleep.

Line 291 in **Figure 1.** predicted odds and confidence bands of self-reported sleep from the fitted multivariate regression model in the primary analysis.

Line 321 in **Figure 2**. Summary of the effect predictors in a fitted linear regression model with a sleep as continues outcome.

Line 338 in **Supp Table 1.** Estimated odds ratios (95% Cis), z-value, p-value from 100 multiple imputations using Rubin’s rules.

- ***Moreover, reviewer comment was “would have like to have seen a regression.”, “In any case, I find multinomial regression extremely complicated to explain and I generally try to avoid it. Given this data and the authors’ aims, I would have thought some sort of linear or non-parametric regression would be easier to explain.”***

We thank the reviewer for this comment and totally agree that a multivariate regression analysis should be used. However, kindly note, similar comments were addressed in an earlier point-by-point response to reviewers which was submitted on 12th October 2023. Please refer to section 1.e. The following were illustrated during that response. “Therefore, to examine the factors associated with self-reported sleep, univariate and multivariate logistic regression models were performed with the dichotomized self-reported sleep as the outcome. The predictors were Total PHQ-8 (linear), Age (linear), Gender (Females vs. Males), BMI (linear), and Marital status (categorical), employment status (categorical). Moreover, a multivariate imputation by chained equations (MICE) procedure was applied with classification and Regression Trees (CART) to impute missing values.”

Kindly refer to line **216** in statistical analysis section, sensitivity analysis in line **239** and (**Table 1 in line 265 & Table 2 in line 282, Figure 1 in line 291 and Figure 2 in 323**) **in the Revised Article with Changes Highlighted**.

**3.c. Reviewer #1 comment:**

***Finally, there are some typos in the text, for example in the abstract the numbers of males and females are not included, it says “n males and n females” (page 4 line 27).***

**Authors response:**

We thank the reviewer for their comment. However, this typo was already edited by authors and submitted during the first revision cycle on 12th October 2023. Also, in the second revision cycle this typo wasn’t present.

**3.d. Reviewer #2 comment:**

***• In the abstract, the reports of conclusions do not seem aligned with the objective and the title. The aim in the abstract/introduction is to investigate the relationship between self-reported sleep hours and self-perceived depression adjusting for sociodemographic factors, but analyses, discussion, and conclusions seem disconnected from this objective.
• In the abstract, there are also several findings summarized in the conclusions that were not even reported in the results section.***

**Authors response:**

We appreciate the reviewer’s comment. However, similar comments were addressed by the initial revision cycle. Kindly refer to sections 1.b and 3.a. Regarding reviewer comment *“In the abstract, there are also several findings summarized in the conclusions that were not even reported in the results section.”,*

Moreover, authors considered edits previously based on previous reviewers’ comments, but also further edits done, and conclusion was re-phrased in order to summarize the findings which serve the main aim and its objectives in a dynamic way. Please refer to line 505 of the conclusion section **in the Revised Article with Changes Highlighted**.

**3.e. Reviewer #2 comment:**

***• The introduction suggests sleep duration as a risk factor of depression, but it is unclear why this paper “focuses on sleep duration as effect of self-perceived depression (lines 62-63)”. Also, due to the cross-sectional study design, the paper can only analyze the association between sleep duration and depression- causal inference cannot be determined.
• Introduction/description of confounders (e.g., age, sex, BMI) in the association between depression and sleep is confusing. For these factors to be confounders, authors need to explain clearly based on the current evidence that they are associated with both the outcome and the exposure, but not on the causal pathway.***

- ***The use of ‘interactive relationship’ and ‘interaction’ in the introduction is also confusing.***

**Authors response:**

We thank the reviewer for their comment. Kindly note these points were addressed previously. Similar comments were brought by the initial revision cycle. Kindly refer to sections **1.b, 1.c, 1.d and 3.a** of this letter.

Also, please find where the sociodemographic factors were explained clearly in the introduction ***are associated with both the outcome and the exposure. Please refer to paragraphs in lines 83, 95 & 100* in the Revised Article with Changes Highlighted*.***

Regarding the *“interaction*”. We thank the reviewer for this important notice, therefore, kindly note the phrase interaction was replaced by association. Please see line 102 in the introduction section **in the Revised Article with Changes Highlighted**.

**3.f. Reviewer #2 comment:**

***Discussion should be written better and be compared with the existing literature.***

**Authors response:**

We thank the reviewer for crucial comments and observation. Although a similar comment was addressed by previous reviewers’ comments, (please see section **1.b and 3.d** of this letter), but we find it very important to meet the recent reviewer comment. Therefore, authors did further edits with supporting literature which has improved the dynamic representation of the paper and presented stronger reflection of the aim and findings and related justification and recommendations. Please refer to line **421 until 474 in the Revised Article with Changes Highlighted.** Moreover, it included justification for some decisions made during data analysis with the support from literature.

**Here are some references for the convenience of the reviewer:**

- Grandner, M. A., Schopfer, E. A., Sands‐Lincoln, M., Jackson, N., & Malhotra, A. (2015). The relationship between sleep duration and body mass index depends on age. Obesity, 23(12), 2491-2498.‏

- Al Balushi, M., Al Balushi, S., Javaid, S., Leinberger-Jabari, A., Al-Maskari, F., Al-Houqani, M., ... & Ali, R. (2022). Association between depression, happiness, and sleep duration: data from the UAE healthy future pilot study. BMC psychology, 10(1), 235.‏

- Sheehan CM, Frochen SE, Walsemann KM, Ailshire JA. Are US adults reporting less sleep? Findings from sleep duration trends in the National Health Interview Survey, 2004–2017. Sleep. 2019 Feb;42(2): zsy221.

- Abe T, Aoki T, Yata S, Okada M. Sleep duration is significantly associated with carotid artery atherosclerosis incidence in a Japanese population. Atherosclerosis. 2011 Aug 1;217(2):509-13.

- Hirshkowitz, M., Whiton, K., Albert, S.M., Alessi, C., Bruni, O., DonCarlos, L., Hazen, N., Herman, J., Katz, E.S., Kheirandish-Gozal, L. and Neubauer, D.N., 2015. National Sleep Foundation’s sleep time duration recommendations: methodology and results summary. Sleep Health, 1(1), pp.40-43.

- Watson, N.F., Badr, M.S., Belenky, G., Bliwise, D.L., Buxton, O.M., Buysse, D., Dinges, D.F., Gangwisch, J., Grandner, M.A., Kushida, C. and Malhotra, R.K., 2015. Recommended Amount of Sleep for a Healthy Adult: A Joint Consensus Statement of the American Academy of Sleep Medicine and Sleep Research Society. Sleep, 38(6), pp.843-844.

- Patel SR, Zhu X, Storfer-Isser A, Mehra R, Jenny NS, Tracy R, Redline S. Sleep duration and biomarkers of inflammation. Sleep. 2009 Feb 1;32(2):200-4.

- Ren L, Wang T, Seklouli AS, Zhang H, Bouras A. A review on missing values for main challenges and methods. Information Systems. 2023 Aug 11:102268.

**3.g. Reviewer #2 comment:**

***Measures: more information on the outcome/exposure should be provided, e.g., how they were measured and what specific questions were used. Authors should discuss limitations, e.g., cross-sectional study design, sampling strategy, bias in self-reported outcome and exposure.***

**Authors response:**

We thank the reviewer for their important comment. Meeting the comments helped to bring clearer information for the reader about the measures section. So, please find the edits done as per lines from 173 to 200 in measures section **in the Revised Article with Changes Highlighted.** Also, specific questions were added to the measures section and would like to bring the reviewer notice that these were also previously included in the supplementary materials.

“Sleep duration was collected by asking the question “how many hours in total do you sleep including day and night?”…

The Patient Health Questionnaire (PHQ-8) is a valid screening measure for depression [20,23]. The following questions were included in the self-perceived questionnaire; for the last two weeks how often have you been bothered by any of the following problems? Little interest or pleasure in doing things, feeling down, depressed, irritable or hopeless, trouble falling or staying asleep, or sleeping too much, feeling tired or having little energy, poor appetite or overeating, feeling bad about yourself – or that you are a failure or have let yourself or your family down, trouble concentrating on things, such as school work, reading or watching television and moving or speaking so slowly that other people could have noticed? or the opposite – being so fidgety or restless that you have been moving around a lot more than usual. The answers included not at all, several days, more than half the days, and nearly every day…

Marital status groups categorized to (married, single), however, individuals with low frequencies, such as divorced, separated, widow and widower, were categorized as others [50]. Employment status was categorized as (employed, un-employed, and students), However, groups with low frequencies, such as retired, housewife, volunteering and unable to work because of sickness or disability, were categorized as others”…

For the reviewer’s convenience, the added statement is shown below and highlighted in blue **in the Revised Article with Changes Highlighted.**

**Moreover, a Strength and limitations section was added which has addressed clearly these parts of the paper and research. Please see lines starting from 488 in the Revised Article with Changes Highlighted.**

**3.h. Reviewer #2 comment:**

***• Analysis section: labelling variable as ‘linear’ is strange- replace by ‘continuous.***

***• Another concern I have is regarding the treatment of missing values as unknown categories in the regression analysis (lines 247-248).***

**Authors response:**

Thank you for the reviewer for this crucial observation. We have replaced linear by continues. Please refer to **lines 224 to 232** of the revised manuscript which has t**he highlights and tracked changes**.

Also, considering the second reviewer’s comment, we agree with the reviewer, therefore, in order to make it clearer, we have made substantial changes to the statistical section in which missing was written as missing and removed the unknown phrase (see lines **251** in the sensitivity analysis of **the Revised Article with Changes Highlighted**), and also made the amendments to the table 1 (lin**e 265**) in which “unknown” was replaced by “missing” **in the Revised Article with Changes Highlighted**.

**3.I. Reviewer #3 comment:**

***The title may need modification to let readers know first-hand what the study entails. Consider a title like ‘’Sociodemographic predictors of the association between self-reported sleep duration and depression.”***

**Authors response:**

We thank the reviewer for the feedback. We agree with the title and therefore, please find it as edited in the revised manuscript. As we found this suggested title brings more understanding to the aim of this paper, we have again edited it. Thank you.

**3.J. Reviewer #3 comment:**

***Abstract (Page 1): The conclusion part of the abstract should reflect the titles. Consideration may be given only to those socio-demographic factors that correlated positively.

2nd Abstract (Page 4): Under “Result”, there were no values for percentages (%) for males and females. Incomplete statement noted within the third statement.***

**Authors response:**

We thank the reviewer for their comment. Kindly note these points were addressed previously. Similar comments were brought by the initial revision cycle. Kindly refer to sections **1.b,1.c, 3.d**. Moreover, this typo does not exist in the revised manuscript which was re-submitted on 12th October2023. Also, the previous cycle of revision didn’t include this typo.

**3.K. Reviewer #3 comment:**

***Introduction: Line 56; there should be a clear definition of depression. What type of disorder is depression?***

**Authors response:**

We thank the reviewer for their comment. Please note depression was defined within the introduction section, refer to line **86 in the Revised Article with Changes Highlighted.**

**3.L. Reviewer #3 comment:**

***Materials and Method:
Under “Measures”, age was categorized as follows; 18 – 19, 20 -29, 30 – 39, and 40+. What is the reason for using 40+ instead of 40 – 49, 50 – 59 etc.***

**Authors response:**

We thank the reviewer for this comment. We would like to draw the attention of the reviewer that this comment was previously highlighted in a previous revision cycle, please refer **to 1.b, 1.e and 3.g**. sections of this letter. Similar research from the same cohort study used the same as well. That is since the focus of the UAE Health Future Study Cohort is to highlight the majority of Emirati population who formulate 61% of the population who undergo between the age 18 to 40 **in the Revised Article with Changes Highlighted**.

Here are the references for the convenience of the reviewer:

- Al Balushi, M., Al Balushi, S., Javaid, S., Leinberger-Jabari, A., Al-Maskari, F., Al-Houqani, M., ... & Ali, R. (2022). Association between depression, happiness, and sleep duration: data from the UAE healthy future pilot study. BMC psychology, 10(1), 235.‏
- Abdulle A, Alnaeemi A, Aljunaibi A, Al Ali A, Al Saedi K, Al Zaabi E, Oumeziane N, Al Bastaki M, Al-Houqani M, Al Maskari F, Al Dhaheri A. The UAE healthy future study: a pilot for a prospective cohort study of 20,000 United Arab Emirates nationals. BMC Public Health. 2018 Dec;18(1):1-9.
- Al Anouti F, Thomas J, Karras S, El Asswad N. Outdoor Activity in the Daytime, but Not the Nighttime, Predicts Better Mental Health Status During the COVID-19 Curfew in the United Arab Emirates. Frontiers in Public Health. 2022 Apr 4; 10:829362.

**3.M. Reviewer #3 comment:**

***Under “Result””
Table 1: The table is easy to understand by scientists.
Figure 1A, 1B, and 1C: What are the reasons for using boxplot?
Is there any table or figure that shows the relationship between sleep hours and depression?***

**Authors response:**

We appreciate the reviewer comments and agree they are very important to the paper. However, kindly note these comments were previously addressed in a previous revision cycle. Kindly note that reviewer is referring to the initial submission version which was already edited on 16th October submission. Also, statistical analysis was re-done, and boxplots were replaced by tables, and more representative figures. Please refer to section 3.b of this letter.

**3.N. Reviewer #3 comment:**

***Discussion and Conclusion:
The conclusion does not highlight and reflects the title of the paper.***

**Authors response:**

Similarly, we appreciate the reviewer comments and agree they are very important to the paper. However, kindly note these comments were previously addressed in a previous revision cycle.

***In general, we thank the reviewers and the academic editor for the overall comments and valuable recommendations which has drastically improved the quality of our research paper. We feel these amendments have majorly improved the academic value and content of our work.***

**Many thanks in advance and looking forward hearing from you,**

**Corresponding author.**

**2nd April 2024**
